# Dalba Got Back? Use of Dalbavancin for the Treatment of Vertebral Osteomyelitis

**DOI:** 10.1093/ofid/ofae070

**Published:** 2024-02-07

**Authors:** Amber C Streifel, Luke C Strnad, Monica K Sikka, Cara D Varley, Jina Makadia, Ellie Sukerman, Alyse H Douglass, Heather Mayer, Kathleen Young, James S Lewis

**Affiliations:** Department of Pharmacy, Oregon Health & Science University, Portland, Oregon, USA; Department of Medicine, Division of Infectious Diseases, Oregon Health & Science University, Portland, Oregon, USA; School of Public Health, Epidemiology Programs, Oregon Health & Science University and Portland State University, Portland, Oregon, USA; Department of Medicine, Division of Infectious Diseases, Oregon Health & Science University, Portland, Oregon, USA; Department of Medicine, Division of Infectious Diseases, Oregon Health & Science University, Portland, Oregon, USA; School of Public Health, Epidemiology Programs, Oregon Health & Science University and Portland State University, Portland, Oregon, USA; Department of Medicine, Division of Infectious Diseases, Oregon Health & Science University, Portland, Oregon, USA; Department of Medicine, Division of Infectious Diseases, Oregon Health & Science University, Portland, Oregon, USA; Department of Medicine, Division of Infectious Diseases, Oregon Health & Science University, Portland, Oregon, USA; Department of Medicine, Division of Infectious Diseases, Oregon Health & Science University, Portland, Oregon, USA; Department of Medicine, Division of Infectious Diseases, Oregon Health & Science University, Portland, Oregon, USA; Department of Pharmacy, Oregon Health & Science University, Portland, Oregon, USA

**Keywords:** long-acting glycopeptides, OPAT, vertebral osteomyelitis

## Abstract

Data evaluating dalbavancin use for vertebral osteomyelitis remain limited. In our retrospective cohort, 29 of 34 (85.3%) patients completed their dalbavancin course. Adverse reactions occurred for 6 (17.6%) and infection recurrence in 3 (8.8%) within 90 days. Dalbavancin appears to be safe and well-tolerated for vertebral osteomyelitis.

Dalbavancin is approved for treatment of skin and soft-tissue infection but has an extended half-life that allows for weekly dosing, making it an attractive alternative for complex infections when standard-of-care antibiotics are not feasible. Limited published evidence evaluating both the pharmacokinetics and the clinical outcomes of dalbavancin for the treatment of osteomyelitis are now available [1, 2]. The use of dalbavancin for the treatment of vertebral osteomyelitis (VO) is not well described, however. The Infectious Diseases Society of America guidelines for the treatment of VO were last updated in 2015, immediately before Food and Drug Administration approval of dalbavancin, and make no mention of its use for the treatment of VO [3].

Because of increasing rates of hospital admission for complex bacterial infections in persons who use drugs (PWUD) [4], the numerous complications associated with vascular access during outpatient parenteral antimicrobial therapy [5], and the challenges of complex daily oral regimens in this vulnerable population, dalbavancin presents an opportunity to provide 6 weeks of antimicrobial therapy with only 2 doses. Whether 2 doses provide adequate systemic drug exposure for *Staphylococcus aureus* VO, which may require at least 8 weeks of therapy to optimize outcomes, is unclear [6].

## METHODS

We conducted a retrospective cohort study of all patients ≥ 18 years treated for VO who received at least 1 dose of dalbavancin in any setting based on recommendations from our institution's infectious diseases (ID) physicians. We identified our cohort using medication records through SAP BusinessObjects Enterprise Business Intelligence Platform 4.2 (SAP America, Inc., Pennsylvania, USA). Demographic data collected include age, gender, infection characteristics, substance use (including alcohol), and reported injection drug use. VO was defined as the combination of cross-sectional radiographic abnormalities or intraoperative findings compatible with VO plus a clinical diagnosis of VO made by ID physicians after formal consultation. Clinical data and treatment outcomes were gathered by chart review. This study was approved by the Oregon Health & Science University institutional review board.

## RESULTS

### Clinical Data

Thirty-four patients met inclusion criteria. Thirty (88%) had imaging compatible with VO by magnetic resonance imaging, 3 (9%) by computed tomography, and 1 (3%) by direct operative visualization in the absence of cross-sectional imaging. In addition to all patients having magnetic resonance imaging/computed tomography/operative evidence plus ID consult assessment of bacterial VO, 18 (82%) of the 22 with spinal samples obtained via biopsy or operation had either culture (13) or pathologic (5) evidence of bacterial infection. In the 4 patients with spinal samples obtained without evidence of infection, 3 had only cultures performed (no pathology) and in 2 there was antecedent antibiotic exposure. In the 16 patients without spinal cultures or pathology positive for infection, 10 (62.5%) had current or very recent prior (<2 months) positive blood cultures with a suspected pathogen. In the remaining 6 (17.6%) cases, there was a heterogenous combination of a culture positive with a suspected VO pathogen at another site, appropriate clinical response to antimicrobial therapy, or lack of another plausible diagnosis that led ID physicians to make the clinical assessment of VO. For 32 patients (94%), this was the first course of dalbavancin they received for the treatment of any infection. Eleven (32.4%) patients were prescribed 1 dose of dalbavancin 1500 mg to complete the approximate equivalent of a 6-week course of antibiotics when combined with previously received antibiotics; 23 (67.6%) patients were prescribed two 1500-mg doses administered 1 week apart following initial antibiotics to result in at least 6 weeks of antimicrobial therapy (Table 1). Mean age was 43.6 years (range, 23–65 years) and 10 (29%) were female. Twenty-seven (79.4%) patients had a documented history of substance use, 23 (67.6%) reported injection drug use, 8 (24%) had history of prior VO, and in 4 (12%) their VO was considered by ID assessment to be a relapse of a recent VO episode. Infections were most commonly from *S aureus* (22; 65%), 10 (45%) of which were methicillin-sensitive *S aureus* (MSSA) and 12 (55%) were from methicillin-resistant *S aureus*. Eleven (32.4%) patients were bacteremic on hospital presentation during this episode of care. Twenty-two patients (64.7%) had epidural space involvement (phlegmon or abscess). Fifteen patients (44.1%) had a source control procedure performed during hospital admission. Retained spinal hardware was present in 8 (23.5%) patients at the time of treatment, although in 5 (14.7%), it was new hardware placed in the source control procedure to address the infection. The recommending ID physician cited injection drug use (24; 70.6%), lack of a safe home environment in which to receive daily IV antibiotics (6; 17.6%), and prior nonadherence to outpatient antibiotics (6; 17.6%) as the most common reasons for selection of dalbavancin. The majority of dalbavancin doses was administered inpatient before hospital discharge or through a home infusion vendor. In 6 (17.6%) cases, chronic suppressive oral antibiotics were recommended after the completion of the dalbavancin course.

**Table 1.** Patient and Infection Characteristics

Planned Dalbavancin Regimen (n = 34)	1500 mg × 1 n = 11	1500 mg × 2 n = 23	All Patients n = 34
Age (y); mean (SD)	48.1 (12.0)	41.5 (10.3)	43.6 (11.2)
	n (%)	n (%)	n (%)
Sex (female)	5 (45.5%)	5 (21.7%)	10 (29.4)
History of substance use	10 (90.9%)	17 (73.9%)	27 (79.4)
Endorsed injection drug use	10 (90.9%)	13 (56.5%)	23 (67.6)

Treatment setting	Single-dose regimens	2 dose regimens		
		First dose	Second dose	
Inpatient	5	12	2	17
Infusion center	1	4	6	7
Home infusion vendor	5	7	9	15
Emergency department	0	0	1	1
Other facility	0	0	1	1

Organism	n (%)	n (%)	n (%)
MRSA	5 (45.5)	7 (30.4)	12 (35.2)
MSSA	4 (36.3)	6 (26.1)	10 (29.4)
*Enterococcus spp.*	1 (9.1)	0	1 (2.9)
*Corynebacterium spp.*+ gram negative	0	1 (4.3)	1 (2.9)
No cultures done	0	3 (13.0)	3 (8.8)
Cultures negative	1 (9.1)	6 (26.1)	7 (20.6)
Infection characteristics			
Bacteremia	6 (54.5)	5 (21.7)	11 (32.4)
Median bacteremia duration; d (range)	2.5 (1–9)	1 (1–18)	2 (1–18)
Epidural space involvement (phlegmon or abscess)	7 (63.4)	15 (65.2)	22 (64.7)
Surgical source control procedure	6 (54.5)	8 (34.8)	14 (41.2)
New spinal hardware placed into infected field	2 (18.2)	1 (4.3)	3 (8.8)
Infected and retained spinal hardware	2 (18.2)	3 (13.0)	5 (14.7)
Median duration of antibiotics before dalbavancin initiation (range)	37 (28–50)	10 (0–30)	18 (0–50)
Concurrent gram-negative antibiotic coverage during dalbavancin course	2 (18.2)	3 (13.0)	5 (14.7)

Documented reason for dalbavancin selection^a^	n (%)
History of IV drug use	24 (70.6)
Substance use, not IV	2 (5.9)
Lack of safe home environment in which to receive daily IV antibiotics	6 (17.6)
Prior nonadherence to outpatient antibiotics	6 (17.6)
Patient refused PICC or daily outpatient IV antibiotics	4 (11.8)
History of contaminated or manipulated PICC	2 (5.9)
Adverse reaction to initial outpatient antibiotic	2 (5.9)
Clinical contraindications to alternative antibiotic options	1 (2.9)
Lack of outpatient options because of funding or insurance limitations	1 (2.9)
Ease of use	1 (2.9)

Abbreviations: IV, intravenous; SD, standard deviation; MRSA, methicillin-resistant Staphylococcus aureus; MSSA, methicillin-sensitive Staphylococcus aureus; PICC, peripherally inserted central catheter.

^a^All reasons documented; individual patients may have more than 1 reason.

### Treatment Outcomes

Ten (90.1%) patients planned for a single dose of dalbavancin and 19 (82.6%) patients planned for 2 doses completed their antibiotic treatment (Table 2). In the single-dose group, the patient who did not complete therapy had the dose initiated but developed an infusion reaction and declined to proceed with the remainder of the dose. Adverse effects are detailed further later. A follow-up visit before the end of therapy was recommended by ID for 31 (91.2%) patients. Of 27 patients scheduled for ID visits, 12 (44.4%) ultimately attended a visit. Recurrence or relapse of infection 30 days after last dalbavancin dose occurred in 2 (5.8%) patients. The causative organisms in these cases were MSSA and methicillin-resistant *S aureus*, respectively. Both patients were intended to receive 2 doses of dalbavancin 1500 mg; 1 completed both doses with progression on imaging adjudicated to be compatible with treatment nonresponse. The other was sent to the emergency department for evaluation of encephalopathy and severe back pain before receiving the second dose of dalbavancin and resuming treatment with standard-of-care antibiotics. Relapse or recurrence at 90 days occurred for 1 (2.9%) patient, who was initially treated empirically with no organism identified. The patient completed 1 of 2 planned doses of dalbavancin and re-presented 3 months later with recrudescence of symptoms, ongoing imaging abnormalities, and a blood culture growing MSSA. Although a surgical intervention for biopsy showed no growth on operative cultures, the situation was assessed by the ID physicians to most likely represent smoldering VO. We observed no deaths at 90-day follow-up. Adverse reactions occurred in 6 (17.6%) patients, with 5 (14.7%) reporting various infusion reactions, including joint pain, flushing, and itching, which resolved with either stopping or slowing infusion and none of which required hospitalization. One (2.9%) reported gastrointestinal upset following dalbavancin infusion.

**Table 2.** Treatment Outcomes

	1500 mg × 1	1500 mg × 2	All Patients
	n (%)	n (%)	n (%)
Completed planned dalbavancin course	10^a^ (90.1)	19 (82.6)	29 (85.3)
ID follow-up			
No follow-up visit recommended in OPAT referral	2 (18.2)	1 (4.3)	3 (8.8)
Visit recommended but not scheduled	2 (18.2)	2 (8.7)	4 (11.8)
Appointment scheduled before end of therapy	7 (63.6)	20 (86.9)	27 (79.4)
Attended as scheduled	5 (71.4)	6 (30.0)	11 (40.7)
Attended after rescheduling	0	1 (5.0)	1 (3.7)
No, missed appointment and lost to follow-up	2 (28.6)	12 (60.0)	14 (51.9)
No, missed appointment because of hospital admission or ED visit	0	1 (5.0)	1 (3.7)
Treatment outcomes			
Chronic oral antibiotic suppression after dalbavancin	2 (18.2)	4 (17.4)	6 (17.6)
Confirmed 30-d readmission for any reason	1 (9.1)	1 (4.3)	2 (5.9)
Confirmed 90-d readmission for any reason	0	2 (8.7)	2 (5.9)
Any readmission by day 90 because of recurrence or adverse effects	0	2 (8.7)	2 (5.9)
Confirmed recurrence or relapse of infection 30-d after last dalbavancin dose (evidence of recurrence or relapse noted in chart)	0	2^b^ (9.1)	2 (5.9)
Confirmed recurrence or relapse of infection 90-d after last dalbavancin dose	0	1^c^ (4.5)	1 (3.7)
Adverse reaction	2 (18.2)	4 (17.4)	6 (17.6)
Gastrointestinal symptoms	1	1	2
Infusion reaction	1	3	4

Abbreviations: ED, emergency department; ID, infectious disease; OPAT, outpatient parenteral antimicrobial therapy.

^a^For 1 patient, infusion was started but not completed because of an infusion reaction.

^b^1 patient completed only 1 of 2 doses; the other patient received 2 doses.

^c^Completed only 1 of 2 doses.

## DISCUSSION AND CONCLUSION

Our real-world data suggest dalbavancin is feasible, well tolerated, and possibly effective as part of a treatment regimen for VO. The majority of cases (22; 64.7%) were due to *S aureus.* The majority of patients had a history of substance use. Completion of the planned dalbavancin course was frequent (29; 85.3%) and relapse or recurrence in patients who completed their planned course of dalbavancin was low (1; 2.9%) compared with literature reporting recurrence rates following treatment of VO with standard-of-care antibiotics (2.2% low risk × 8 weeks; 34.8% high risk × 6 weeks) [6]. However, loss to follow-up occurred in 14 (41.2%) patients who had a follow-up visit scheduled, so some cases of relapse or recurrence may have gone undetected. If the worst-case scenario is assumed that all patients lost to follow-up had a relapse or recurrence within 90 days of treatment, our failure rate is approximately 47%, which is higher than rates in the general population treated with standard-of-care antibiotics; this highlights how it is difficult to draw strong conclusions about clinical outcomes without more complete follow-up data.

The data supporting the use of dalbavancin for the treatment of osteomyelitis are primarily pharmacokinetic, showing sufficient target attainment in serum and bone to at least 6 weeks following 2 doses of 1500 mg administered 1 week apart [1]. Case series have documented the tolerability of dalbavancin for the treatment of osteomyelitis, even when more than 2 doses were received [7]. A retrospective observational cohort study identified no difference between dalbavancin and standard-of-care antibiotics in rates of treatment failure, readmission, or adverse events for the treatment of osteomyelitis [8]. An 80-patient randomized control study comparing dalbavancin with vancomycin with a randomization of 7:1 demonstrated dalbavancin to be noninferior for patients with first occurrence of osteomyelitis who received baseline debridement and did not have hardware present at the site of infection [2]. In contrast, in our cohort, fewer than half of patients had a source control procedure, despite a high rate of epidural involvement, but still had promising outcomes acknowledging the high rate of those lost to follow-up. In the ongoing randomized controlled trial (DOTS) evaluating dalbavancin in patients with complicated *S aureus* bacteremia or endocarditis, epidural space involvement was excluded, so this much-awaited data still will not elucidate the utility of dalbavancin in cases complicated by VO [9].

Although case reports and small series describing the use of dalbavancin for vertebral osteomyelitis have been published [10, 11], our sample is the largest published to date in the US healthcare system, where resources and payment structure differ significantly from other countries and result in additional challenges to the use of dalbavancin in the outpatient setting [12]. In addition to long-acting glycopeptides, oral antibiotics offer an alternative to daily intravenous antibiotics and peripherally inserted central catheters in the community. The OVIVA trial included patients with VO, although these cases made up a small portion of the total patients included (< 7%) in the study [13]. The complexity of oral antibiotic regimens, especially for vulnerable populations who may not have a safe storage place for these medications, must be taken into account when considering this alternative to daily intravenous antibiotics.

Given that the majority of patients (79%) in our cohort treated with dalbavancin for VO had reported drug use, it is critical to consider the use of long-acting agents in PWUD. The use of dalbavancin in PWUD has been described in the treatment of various infections to result in cost savings and reduced length of hospital stay [14–16]. PWUD experience significant stigma within the healthcare system [17, 18], including among ID physicians [19], despite data demonstrating successful completion of outpatient parenteral antimicrobial therapy in this patient population [20]. Although long-acting glycopeptides offer appealing alternatives to daily intravenous antibiotics in the outpatient setting, more clinical data are needed to ensure that these infections are being treated optimally for all patients before recommending this as a standard-of-care equivalent to more proven daily intravenous or oral regimens.

### Limitations and Future Research

Our study has a number of limitations. This was a retrospective review reliant on chart notes and documentation to gather treatment information and clinical reasoning, which may not have captured all pertinent details used to select a regimen at the time of initial ID consultation. Our clinical outcomes data are limited by the fact that 22 (64.7%) patients either were not recommended for follow-up in a clinic or ultimately did not attend a follow-up visit. Additionally, readmission and recurrence data are limited to presentation at our institution and do not capture readmission or recurrence treated at outside hospitals. Future research should include prospective and randomized trials to establish dalbavancin's general place in therapy for the treatment of VO compared to current standard-of-care intravenous antibiotics and oral antibiotics, as well as the use of these therapies in populations with substance use disorder.

### Conclusion

Dalbavancin offers a potentially promising alternative to more complicated antibiotic regimens for the treatment of VO. The broad gram-positive activity, ease of administration, and tolerable side effect profile create unique advantages for this and similar agents. However more clinical data are needed to truly determine its place in VO therapy, in particular ideal duration of other antibiotics before the transition to dalbavancin and in the treatment of VO caused by *S aureus,* in which more than 6 weeks of antibiotic therapy is often needed.

## Notes

***Acknowledgments.*** Sir-Mix-a-Lot, Ryan Markham.

***Patient consent statement***. The OHSU IRB approved this study (STUDY00019068) and approved a waiver of consent.
